# Differences in serum SP-D levels between German and Japanese subjects are associated with *SFTPD* gene polymorphisms

**DOI:** 10.1186/1471-2350-15-4

**Published:** 2014-01-08

**Authors:** Yasushi Horimasu, Noboru Hattori, Nobuhisa Ishikawa, Sonosuke Tanaka, Francesco Bonella, Shinichiro Ohshimo, Josune Guzman, Ulrich Costabel, Nobuoki Kohno

**Affiliations:** 1 Department of Molecular and Internal Medicine, Graduate School of Biomedical Science, Hiroshima University, 1-2-3 Kasumi, Minami-ku, Hiroshima 734-8551, Japan; 2 Department of Pneumology/Allergy, Ruhrlandklinik, University Hospital, University Duisburg-Essen, Tueschener Weg 40, 45239 Essen, Germany; 3 General and Experimental Pathology, Ruhr University Bochum, Universitätsstraße 150, 44801 Bochum, Germany

**Keywords:** Biological marker, Idiopathic interstitial pneumonia, Single nucleotide polymorphism, Surfactant protein-A (SP-A), Surfactant protein-D (SP-D)

## Abstract

**Background:**

Surfactant protein A (SP-A) and SP-D are clinically established in Japan as serum biomarkers for diagnosing interstitial lung diseases (ILDs). Serum SP-D levels are affected by genetic variants. We conducted the present study to examine whether serum SP-A and/or SP-D levels in healthy subjects (HS) and patients with ILDs differ between populations with different genetic backgrounds.

**Methods:**

German subjects (n = 303; 138 patients with idiopathic interstitial pneumonias [IIPs] and 165 HS) and Japanese subjects (n = 369; 94 patients with IIPs and 275 HS) were enrolled. Serum SP-A and SP-D levels were measured using an enzyme-linked immunosorbent assay, and four single-nucleotide polymorphisms (SNPs) in the *SFTPD* gene were genotyped using genomic DNA extracted from blood samples.

**Results:**

In both the German and Japanese cohorts, serum SP-A and SP-D levels were significantly higher in patients with IIPs than in HS. There were no significant differences in SP-A levels between the German and Japanese cohorts; however, we found that serum SP-D levels were significantly higher in the German cohort, both in patients with IIPs and in HS (*p* < 0.001 and *p* = 0.005, respectively). Furthermore, the genotype distributions of the four SNPs in the *SFTPD* gene (rs721917, rs1998374, rs2243639, and rs3088308) were significantly different between German and Japanese cohorts (*p* < 0.001, *p <* 0.001, *p =* 0.022, and *p* < 0.001, respectively), and univariate linear regression analyses revealed that the genotypes of rs721917, rs1998374, and rs2243639 significantly correlated with serum SP-D levels (*p* < 0.001, *p* < 0.001, and *p* = 0.011, respectively). Furthermore, multivariate analyses revealed that the genotypes of these three SNPs correlated independently with serum SP-D levels (*p* < 0.001, *p* = 0.001, and *p* = 0.038, respectively), whereas ethnicity did not significantly correlate with serum SP-D levels.

**Conclusions:**

In patients with IIPs and HS, serum SP-D, but not SP-A, levels were significantly higher in the German than in the Japanese cohort, in part, because of the different frequencies of *SFTPD* gene polymorphisms.

## Background

Idiopathic interstitial pneumonias (IIPs) are a group of diffuse parenchymal lung diseases characterized by interstitial involvement resulting from various patterns of inflammation and fibrosis of unknown cause. The prevalence of IIPs has been generally reported as 5–20 per 100,000 persons [1-4], with a recommendation that the diagnosis of IIPs be made according to clinical history, physical findings, chest radiographs, and/or lung function tests [5,6]. However, some patients may not complain of symptoms or present with abnormal chest radiographs and/or lung function tests, even though they already suffer from IIPs. Therefore, the availability of diagnostic tools that can discriminate patients with IIPs from healthy subjects (HS) at an early stage will be undoubtedly useful in clinical practice. In this regard, serum biomarkers draw particular interest because they are easy to obtain from patients.

Pulmonary surfactant protein A (SP-A) and SP-D are water-soluble proteins derived mainly from type II pneumocytes and belong to the collectin subgroup of the C-type lectin superfamily [7]. Because one of the key histological feature of the lung affected with interstitial lung diseases (ILDs) involves injury and/or regeneration of Type II pneumocytes [8], soluble proteins derived from Type II pneumocytes, such as SP-A, SP-D, and Krebs von den Lungen 6 (KL-6), have been studied as potential biomarkers for ILDs [9-15]. These biomarkers can be useful for early detection of ILDs, predicting disease outcome, and monitoring the clinical course [16-18]. On the basis of these findings, serum SP-A, SP-D, and KL-6 have been clinically approved by Japan’s Health Insurance Program as diagnostic markers for ILDs in 1999, and more than 2,000,000 samples of these biomarkers are now examined yearly in Japan. However, in most countries, assays for these biomarkers are limited to research and are currently unavailable for clinical practice.

We recently conducted an international study to measure the serum levels of KL-6 and analyze the rs4072037 genotypes of Mucin 1 (*MUC1*) in German and Japanese cohorts that included patients with ILDs and healthy subjects. We demonstrated that the cutoff value of KL-6 that discriminated patients with ILDs from HS was significantly higher in the German than in the Japanese cohort because of differences in the distribution of rs4072037 genotypes between them [19]. The correlations between rs4072037 genotypes and serum KL-6 levels have also been demonstrated in a Dutch cohort [20]. Moreover, serum SP-D levels were found to be correlated with genetic polymorphisms of surfactant protein D (*SFTPD*) [21-23]. According to the International HapMap project [24], the genotype distributions of some single-nucleotide polymorphisms (SNPs) in the *SFTPD* gene differ according to ethnicities. We hypothesized, therefore, that differences exist in serum SP-D and/or SP-A levels between different ethnic populations. To test this hypothesis, we first measured serum SP-A and SP-D levels and compared them between German and Japanese cohorts that included patients with IIPs and healthy subjects. Next, we evaluated the correlations between serum SP-D levels and *SFTPD* gene polymorphisms in the German and Japanese cohorts.

## Methods

### Study subjects

Between February 2007 and December 2011, 138 consecutive German-Caucasian patients with IIPs at Ruhrlandklinik, University Hospital Essen (Essen, Germany) and 94 Japanese patients with IIPs at Hiroshima University Hospital (Hiroshima, Japan) were enrolled in the present study. 165 German-Caucasian and 275 Japanese HS were recruited from the subjects who visited these hospitals to undergo health checkup. Diagnoses of IIPs were made according to the criteria of the American Thoracic Society (ATS)/European Respiratory Society (ERS) published in 2002 [5]. We excluded patients with ILDs of known cause, including drugs, collagen vascular diseases, and hypersensitivity pneumonia. Each HS underwent pulmonary function tests and chest X-ray studies, and we excluded those with apparent lung disease such as ILDs or chronic obstructive pulmonary disease (COPD). The Ethics Committees of Ruhrlandklinik (IRB 06-3170) and Hiroshima University Hospital (IRB 326) approved this study, which was conducted in accordance with the ethical standards established by the Helsinki Declaration of 1975. All patients and healthy volunteers provided written informed consent to participate in the study and permission to use their samples.

### Lung function values

Physiologic assessment included measurements of thoracic gas volume, total lung capacity, forced vital capacity (FVC), forced expiratory volume in one second (FEV1), and single-breath diffusing capacity of the lung for carbon monoxide (DL_CO_) as previously described [25-28]. The protocol for lung function measurements conformed to ATS recommendations [29].

### Measurement of serum SP-A and SP-D levels

Serum samples were obtained from 234 patients with IIPs and 440 HS and stored at -80°C. Serum SP-A and SP-D levels were measured using a sandwich-type enzyme-linked immunosorbent assay (ELISA) by using commercially available ELISA kits (SP-A test Kokusai-F kit, Sysmex Corporation Kobe, Japan; SP-D kit YAMASA EIA II, Yamasa Corporation Choshi, Japan) as previously described [28,30].

### DNA preparation and SNP genotyping

**Figure 1:**
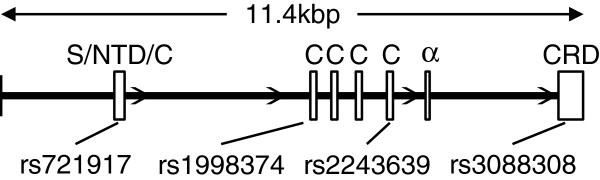
**Functional features of the *SFTPD *gene.** The thick horizontal bar represents the intronic region, and the white boxes represent exons. S, signal peptide; NTD, N-terminal domain; C, collagen domain; α, α-helical neck region; CRD, carbohydrate recognition domain.

We extracted DNA from peripheral whole venous blood samples by using the phenol-chloroform extraction and ethanol precipitation methods as previously described [31]. SNPs only in the *SFTPD* gene were investigated because difference in serum levels between German and Japanese was just observed for SP-D but not for SP-A. We analyzed HapMap genotyping data [24] by using chi-square tests and picked up 18 SNPs in the *SFTPD* gene that showed significantly different genotype distributions between CEU (Utah residents with ancestry from northern and western Europe) and JPT (Japanese in Tokyo, Japan). From these 18 SNPs, we selected four SNPs (rs721917, rs1998374, rs2243639, and rs3088308; Figure 1) that have been reported to be associated with serum SP-D levels [21-23] and/or TaqMan SNP Genotyping Assays are available for. SNP genotyping was carried out by using TaqMan SNP Genotyping Assay C 1362980-10, C 12124514-10, C 26726205-10, and C 26726209-10 (Life Technologies Corp. Carlsbad, California, USA) and the Applied Biosystems 7500 Fast Real-Time PCR System (Life Technologies Corp. Carlsbad, California, USA).

### Statistical analysis

Data are presented as the mean ± standard error of the mean (SEM). Data for individual variables for 2 groups were analyzed using the Mann–Whitney *U*-test or the chi-square test. The significance levels for multiple pairwise comparisons were set according to Bonferroni’s correction. Receiver operating characteristic (ROC) curves were generated to assess the diagnostic abilities of SP-A and SP-D. Linear regression analyses were conducted to study the correlations between serum SP-D levels and each of the four SNPs. If the correlation was statistically significant, multivariate regression analysis was performed to determine whether the SNPs in the *SFTD* gene independently affect serum SP-D levels even when adjusted by the covariates as follows: age, ethnicity, and case-control status. All statistical analyses were performed using SPSS for Windows, version 18.0 (SPSS Inc. Chicago, USA).

## Results

### Serum SP-D but not SP-A levels were significantly higher in the German cohort

**Figure 2:**
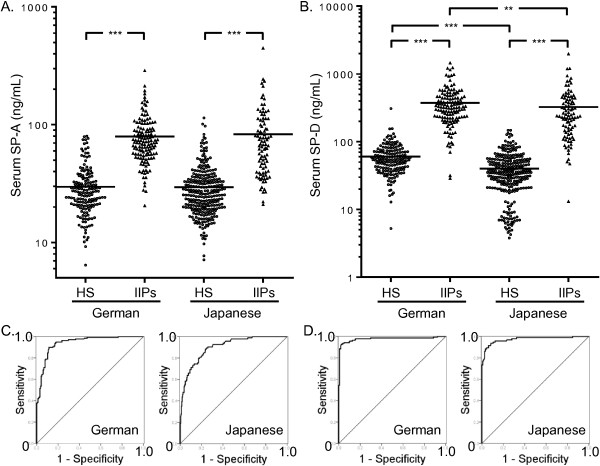
**Comparison of serum SP-A and SP-D levels between the cohorts. (A)** Serum SP-A and **(B)** SP-D levels in German and Japanese cohorts. Receiver operating characteristic (ROC) curves of **(C)** serum SP-A and **(D)** SP-D in German (left panel) and Japanese (right panel) cohorts. The horizontal bars represent the mean values. The significance level was defined α = 0.013 (four comparisons in four groups). ***p < 0.001, **p < 0.013 (Mann–Whitney *U*-test).

The clinical characteristics of 232 patients with IIPs (138 German and 94 Japanese) and 440 HS (165 German and 275 Japanese) are shown in Table 1. Serum SP-A levels in German HS, German patients with IIPs, Japanese HS, and Japanese patients with IIPs were 29.7 ± 1.1 ng/ml, 79.4 ± 3.3 ng/ml, 29.4 ± 0.9 ng/ml, and 83.0 ± 6.3 ng/ml, respectively (Figure 2A). Serum SP-D levels in German HS, German patients with IIPs, Japanese HS, and Japanese patients with IIPs were 59.8 ± 2.6 ng/ml, 373.3 ± 20.3 ng/ml, 39.9 ± 1.6 ng/ml, and 323.9 ± 29.8 ng/ml, respectively (Figure 2B). In both the German and the Japanese cohort, the serum SP-A and SP-D levels in patients with IIPs were significantly higher than those in HS. Further, serum SP-D levels in German HS were significantly higher than those in Japanese HS (*p* < 0.001; Figure 2B), and serum SP-D levels in German patients with IIPs were significantly higher than those in Japanese patients with IIPs (*p* = 0.005; Figure 2B). In contrast, there was no significant difference in serum SP-A levels between the cohorts, within the HS and patients with IIPs (*p* = 0.677 for HS and *p* = 0.326 for patients with IIPs; Figure 2A).

**Table 1:** Characteristics of study subjects

	**German**	**Japanese**	***p *** **value**
**Patients with IIPs**			
Number of the subjects	138	94	
Age (years)	67.4 ± 0.8	68.0 ± 1.0	0.982
Gender (male/female)	88 (63.8%)/50 (36.2%)	64 (68.1%)/30 (31.9%)	0.497
Smoking (Cu/Ex/Non/ND)	14 (10.1%)/54 (39.1%)/	11 (11.7%)/46 (49.0%)/	0.297
	64 (46.4%)/6 (4.4%)	35 (37.2%)/2 (2.1%)	
VC (percent predicted)	66.1 ± 1.6	70.3 ± 2.2	0.163
DL_CO_ (percent predicted)	47.7 ± 1.6	44.8 ± 1.8	0.255
Diagnostic category (IPF/NSIP)	94 (68.1%)/44 (31.9%)	61 (64.9%)/33 (35.1%)	0.609
**Healthy subjects**			
Number of the subjects	165	275	
Age (years)	36.5 ± 0.9	49.8 ± 0.4	< 0.001
Gender (male/female)	60 (36.4%)/105 (63.6%)	227 (82.5%)/48 (17.5%)	< 0.001
Smoking (Cu/Ex/Non/ND)	41 (24.8%)/20 (12.1%)/	82 (29.8%)/62 (22.6%)/	0.026
	90 (54.6%)/14 (8.5%)	131 (47.6%)/0 (0.0%)	

Data are shown as mean ± SEM.

Statistical significance was tested by Mann–Whitney *U*-test or Chi-square test.

*Cu*, Current smoker; *Ex*, Ex-smoker; *Non*, Non-smoker; *ND*, No data; *IIPs*, Idiopathic interstitial pneumonias; *VC*, Vital capacity; *DL*_*CO*_, Diffusing capacity of the lung for carbon monoxide; *IPF*, Idiopathic pulmonary fibrosis; *NSIP*, Nonspecific interstitial pneumonia.

To evaluate the ability of the serum levels of SP-A and SP-D to discriminate patients with IIPs from HS, ROC curves were drawn for each cohort. The areas under the curves (AUCs) for serum SP-A in the German (AUC = 0.940, 95% confidence interval [CI] = 0.915–0.966; *p* < 0.001) and Japanese cohorts (AUC = 0.902, 95% CI = 0.868–0.936; *p* < 0.001) were sufficiently high to distinguish patients with IIPs from HS (Figure 2C). This was true for the AUCs for serum SP-D levels in the German (AUC = 0.977, 95% CI = 0.957–0.996; *p* < 0.001) and Japanese cohorts as well (AUC = 0.973, 95% CI = 0.952–0.994; *p* < 0.001; Figure 2D).

### Distributions of *SFTPD* gene polymorphisms are not different between patients with IIPs and healthy subjects

DNA was extracted from blood samples from 139 of 303 German (102 patients with IIPs and 37 HS) and 338 of 369 Japanese subjects (63 patients with IIPs and 275 HS). Because collection of whole blood for DNA extraction was missed or refused, we could not obtain the genotype data in a part of German patients with IIPs, German HS, and Japanese patients with IIPs. We compared subject characteristics including serum SP-A and SP-D levels based on the availability of genotype data and found that there was no significant difference in serum SP-A or SP-D levels between the subjects with and without genotype data in German patients with IIPs, German HS, or Japanese patients with IIPs (Additional file 1: Tables S1A and S1B). The genotype distributions of the four SNPs were in Hardy–Weinberg equilibrium in German patients with IIPs, German HS, Japanese patients with IIPs, and Japanese HS (Table 2). As shown in Table 2, the genotype distributions of the four SNPs did not differ between patients with IIPs and HS in both the German and Japanese cohorts. Further, the four SNPs (rs721917, rs1998374, rs2243639, and rs3088308) showed significantly different genotype distributions between the German and Japanese cohorts (*p* < 0.001, *p <* 0.001, *p =* 0.022, and *p <* 0.001, respectively; Additional file 2: Table S2).

**Table 2:** **Genotype distributions of single nucleotide polymorphisms in *SFTPD *** **gene – patients with IIPs vs HS –**

	**German**					**Japanese**				
**rs721917**	Total	C/C	T/C	T/T	HWE	Total	C/C	T/C	T/T	HWE
Patients with IIPs	102	25	47	30	0.862	63	19	30	14	0.973
		(24.5%)	(46.1%)	(29.4%)			(30.2%)	(47.6%)	(22.2%)	
Healthy subjects	37	9	11	17	0.266	275	95	136	44	0.960
		(24.3%)	(29.7%)	(46.0%)			(34.5%)	(49.5%)	(16.0%)	
Chi-square test		*p* = 0.140					*p* = 0.476			
**rs1998374**	Total	C/C	T/C	T/T	HWE	Total	C/C	T/C	T/T	HWE
Patients with IIPs	102	3	18	81	0.655	63	6	35	22	0.561
		(2.9%)	(17.7%)	(79.4%)			(9.5%)	(55.6%)	(34.9%)	
Healthy subjects	37	0	5	32	0.913	275	57	125	93	0.679
		(0.0%)	(13.5%)	(86.5%)			(20.7%)	(45.5%)	(33.8%)	
Chi-square test		*p* = 0.464					*p* = 0.102			
**rs2243639**	Total	C/C	T/C	T/T	HWE	Total	C/C	T/C	T/T	HWE
Patients with IIPs	102	43	45	14	0.960	63	28	28	7	1.000
		(42.2%)	(44.1%)	(13.7%)			(44.4%)	(44.4%)	(11.1%)	
Healthy subjects	37	13	15	9	0.741	275	140	113	22	0.996
		(35.1%)	(40.6%)	(24.3%)			(50.9%)	(41.1%)	(8.0%)	
Chi-square test		*p* = 0.324					*p* = 0.563			
**rs3088308**	Total	A/A	A/T	T/T	HWE	Total	A/A	A/T	T/T	HWE
Patients with IIPs	102	86	16	0	0.711	63	59	4	0	0.968
		(84.3%)	(15.7%)	(0.0%)			(93.7%)	(6.3%)	(0.0%)	
Healthy subjects	37	32	5	0	0.913	275	265	10	0	0.955
		(86.5%)	(13.5%)	(0.0%)			(96.4%)	(3.6%)	(0.0%)	
Chi-square test		*p* = 0.752					*p* = 0.330			

*IIPs*, Idiopathic interstitial pneumonias; *HWE*, Hardy-Weinberg equilibrium.

### Serum SP-D levels differ according to *SFTPD* gene polymorphisms

**Figure 3:**
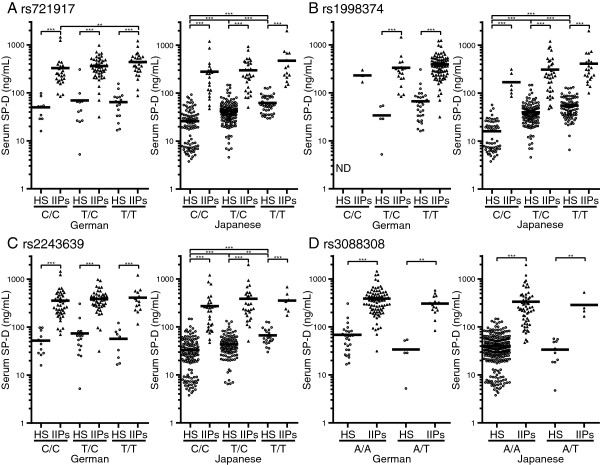
**Relationship between genotype and serum SP-D levels.** Serum SP-D levels were compared between each genotype: **(A)** rs721917, **(B)** rs1998374, **(C)** rs2243639, and **(D)** rs3088308. For each genotype, data of the German cohort are shown in the left and those of the Japanese cohort are shown in the right. The horizontal bars represent the mean values. **(A)**, **(B)**, **(C)** The significance level was defined as α = 0.006 (nine comparisons in six groups). ***p < 0.001, **p < 0.006 (Mann–Whitney *U*-test). **(D)** The significance level was defined as α = 0.0125 (four comparisons in four groups). ***p < 0.001, **p < 0.013 (Mann–Whitney *U*-test).

Within each German and Japanese cohort, serum SP-D levels were compared based on the genotypes of each SNP in the *SFTPD* gene. Within all genotypes of the four SNPs, the serum SP-D levels of patients with IIPs were significantly higher than those of HS in both German and Japanese cohorts (Figure 3). Furthermore, serum SP-D levels of Japanese HS were significantly higher in the rs721917 T/T than in the T/C cohort (*p* < 0.001) and were also significantly higher in the T/C than in the C/C cohort (*p* < 0.001; Figure 3A). Moreover, serum SP-D levels of patients with IIPs tended to be higher in the rs721917 T/T and be lower in the C/C cohort in both German and Japanese populations (Figure 3A). Similarly, serum SP-D levels also showed a trend to be different between the genotypes of rs1998374 (Figure 3B) and rs2243639 (Figure 3C). In contrast, serum SP-D levels did not differ according to the rs3088308 genotypes (Figure 3D).

### Correlation between serum SP-D levels and *SFTPD* gene polymorphisms is statistically independent

Linear regression analyses were performed to further examine whether the *SFTD* gene polymorphisms affect serum SP-D levels independently from other covariates. Univariate analyses revealed that the rs721917, rs1998374, and rs2243639 genotypes correlated significantly with serum SP-D levels (*p* < 0.001, *p* < 0.001, and *p =* 0.011, respectively), but not that of rs3088308 (Table 3). We next performed multivariate analysis, including age, ethnicity, and case-control status (patients with IIPs vs HS) as covariates, because these covariates showed significant correlations with serum SP-D levels in univariate analyses (*p* < 0.001, *p* < 0.001, and *p* < 0.001, respectively). The rs721917, rs1998374, and rs2243639 genotypes significantly correlated with serum SP-D levels in multivariate analyses (*p* < 0.001, *p* = 0.001, and *p* = 0.038, respectively) as well. Further, among the three covariates, only the case-control status revealed significant correlations with serum SP-D levels in the three multivariate models (*p <* 0.001, *p <* 0.001, *p <* 0.001, respectively; Table 3), although age and ethnicity did not significantly correlate with serum SP-D levels.

**Table 3:** **Correlations between serum SP-D levels and *SFTPD *** **gene polymorphisms**

	**Variable**	**Regression coefficient (95% CI.)**	***p *** **value**	**VIF**
**rs721917**	**Univariate model**			
	C/C vs T/C vs T/T	55.276 (28.473 – 82.079)	< 0.001	
	**Multivariate model**			
	C/C vs T/C vs T/T	35.149 (15.341 – 54.957)	< 0.001	1.031
	Age	-0.444 (-2.009 – 1.121)	0.578	1.978
	Japanese vs German	24.061 (-13.125 – 61.246)	0.204	1.437
	HS vs patients with IIPs	307.740 (260.743 – 354.736)	< 0.001	2.515
**rs1998374**	**Univariate model**			
	C/C vs T/C vs T/T	86.550 (59.832 – 113.269)	< 0.001	
	**Multivariate model**			
	C/C vs T/C vs T/T	36.172 (14.289 – 58.056)	0.001	1.203
	Age	-0.353 (-1.918 – 1.213)	0.658	1.972
	Japanese vs German	11.114 (-28.165 – 50.393)	0.578	1.598
	HS vs patients with IIPs	304.645 (257.602 – 351.689)	< 0.001	2.511
**rs2243639**	**Univariate model**			
	C/C vs T/C vs T/T	38.179 (8.965 – 67.393)	0.011	
	**Multivariate model**			
	C/C vs T/C vs T/T	22.707 (1.310 – 44.104)	0.038	1.017
	Age	-0.360 (-0.449 – 0.654)	0.654	1.976
	Japanese vs German	29.314 (-8.000 – 66.627)	0.123	1.423
	HS vs patients with IIPs	306.421 (259.045 – 353.798)	< 0.001	2.514
**rs3088308**	**Univariate model**			
	A/A vs A/T	37.750 (-37.732 – 113.231)	0.326	

The genotypes of each single nucleotide polymorphism are arranged in the order of lowest to highest serum SP-D levels.

*CI*, Confidence interval; *HS*, Healthy subjects; *IIPs*, Idiopathic interstitial pneumonias; *VIF*, Variance inflation factor.

## Discussion

In the present study, we demonstrated that serum SP-A and SP-D levels were significantly higher in patients with IIPs than in HS in both German and Japanese subjects. Moreover, we found that in patients with IIPs and HS, serum SP-D levels were significantly higher in the German than in the Japanese cohort, whereas there were no significant differences in serum SP-A levels between the two cohorts. The genotype distributions of SNPs in the *SFTPD* gene, which affect the serum SP-D levels, differed between the cohorts. Furthermore, multivariate analyses demonstrated that there were statistically independent correlations between serum SP-D levels and the rs721917, rs1998374, and rs2243639 genotypes of the *SFTPD* gene, regardless of ethnicity and presence of IIPs.

The significantly higher serum SP-A and SP-D levels in patients with IIPs compared with those of the German HS suggest their utility as diagnostic biomarkers for IIPs, even in the German population. Serum SP-A levels in the German patients with IIPs or HS were comparable to those in their Japanese counterparts (Figure 2A); however, the serum SP-D levels in German patients with IIPs or HS were significantly higher than those in their Japanese counterparts (Figure 2B). These results imply that serum SP-D levels are affected by the different ethnicity, whereas serum SP-A levels are not. In agreement with our present study results, serum SP-D levels in Caucasian HS were reported to be higher than those in Asian HS [32].

To explain the differences in serum SP-D levels between the German and Japanese cohorts, we determined the relationship between serum SP-D levels and genotypic differences in the *SFTPD* gene. We found that among the SNPs in *SFTPD* gene, rs721917, rs1998374, and rs2243639, but not rs3088308, affected the serum SP-D levels (Figure 3), and the distributions of these polymorphisms were different between the German and Japanese cohorts. SP-A and SP-D molecules comprise an N-terminal domain (NTD), a collagen domain, an α-helical neck region, and a carbohydrate recognition domain (CRD; Figure 1) [33]. SP-D polypeptide chains bind together through interpolypeptide disulfide bonds in the NTD to form oligomers, and the degree of oligomerization is affected by the genotypes of coding SNP rs721917 in the NTD. Thus, the T/T and C/C genotypes correlate with higher and lower-order oligomers, respectively [33-36]. These structural variations of SP-Ds might affect their serum levels, and our results demonstrate that serum SP-D levels differed according to the rs721917 genotype (Figure 3A). Furthermore, we also found that serum SP-D levels were affected by the genotypes of rs1998374 and rs2243639, both of which are located in the collagen domain (Figures 1, 3B and C), but they were not affected by the genotypes of rs3088308, the coding SNP in the CRD (Figures 1 and 3D). These findings suggest the possibility that the collagen domain is also associated with the degree of oligomerization of the SP-D molecule.

To determine whether serum SP-D levels were independently correlated with *SFTPD* gene polymorphisms and/or ethnicity, we performed multivariate regression analyses and found that the correlations between the genotypes of three SNPs in the *SFTPD* gene and serum SP-D levels remained statistically significant in the multivariate models (Table 3). In contrast, the correlations between ethnicity and serum SP-D levels were insignificant (Table 3). These findings suggest that serum SP-D levels are more strongly affected by *SFTPD* gene polymorphisms than by ethnicity. Therefore, the difference in serum SP-D levels that were observed between German and Japanese cohorts might be partially explained by the differences in the frequencies of *SFTPD* gene polymorphisms between the cohorts.

In contrast, we found that the genotype distributions of four SNPs in the *SFTPD* gene did not differ between patients with IIPs and HS in both cohorts (Table 2). SP-A and SP-D play important roles in surfactant-related functions and in host defense against inhaled pathogens [37]. Moreover, *SFTPD* gene polymorphisms, rs721917 in particular, have been reported to correlate with susceptibility to COPD, community-acquired pneumonia, ILDs, and lung cancer [23,38-41]. As discussed above, rs721917 is known to be associated with the degree of oligomerization of the SP-D molecules [33-36]. This difference in oligomerization might affect the surfactant and/or host defense functions of SP-D and thus correlate with susceptibility to various respiratory diseases. In the present study, however, no significant correlation between *SFTPD* gene polymorphisms and susceptibility to IIPs was demonstrated. We believe that a larger sample size of study is needed to determine the correlations between *SFTPD* gene polymorphisms and susceptibility to IIPs.

We are aware that there are some limitations in this study. First, age, gender, and smoking status were significantly different between German HS and Japanese HS because the populations who undergo health checkup were different between Germany and Japan. We performed linear regression analysis to assess the interference between these factors and serum SP-D levels, and we found a significant correlation between age and serum SP-D levels. Thus, we included age into the multivariate analyses and confirmed that our results were significant and independent of age differences. Second, the number of subjects available for genomic analyses was relatively small. Third, only German and Japanese populations were studied. It remains unclear whether the findings of the present study can be applied to other ethnic groups such as African Americans.

## Conclusions

In conclusion, we demonstrated that the serum levels of SP-A and SP-D were significantly elevated in patients with IIPs in the German and Japanese cohorts, whereas serum SP-D but not SP-A levels were significantly higher in the German cohort. We have explained the differences in serum SP-D levels between these cohorts, at least in part, by the different frequencies of *SFTPD* gene polymorphisms. Although we believe that these data are compelling, further investigations with larger number of subjects are required to assess the utility of serum SP-A and SP-D in non-Japanese cohorts.

## Abbreviations

IIP: Idiopathic interstitial pneumonia; SP-A: Surfactant protein A; SP-D: Surfactant protein D; ILD: Interstitial lung disease; KL-6: Krebs von den Lungen 6; MUC1: Mucin 1; SFTPD: Surfactant protein D; SNP: Single nucleotide polymorphism; HS: Healthy subjects; ATS: American Thoracic Society; ERS: European Respiratory Society; COPD: Chronic obstructive pulmonary disease; FVC: Forced vital capacity; FEV1: Forced expiratory volume in one second; DLCO: Single-breath diffusing capacity of the lung for carbon monoxide; ELISA: Enzyme-linked immunosorbent assay; ROC: Receiver operating characteristic; AUC: Area under the curve; CI: Confidence interval; NTD: N-terminal domain; CRD: Carbohydrate recognition domain.

## Competing interests

The authors declare that they have no competing interests.

## Authors’ contributions

YH drafted and finalized the manuscript, performed part of the serum measurement, extraction of DNA, genotyping, and statistical analyses. NH, NI, NK and UC conceived the study, and participated in its design and coordination and helped to draft and finalize the manuscript. ST performed part of the extraction of DNA and genotyping. FB, JG and SO recruited the study subjects, ascertained diagnosis, and helped to draft and finalize the manuscript. All authors read and approved the final manuscript.

## Pre-publication history

The pre-publication history for this paper can be accessed here:

http://www.biomedcentral.com/1471-2350/15/4/prepub

## Supplementary Material

Additional file 1: Table S1 Comparisons of the baseline characteristics including serum SP-A and SP-D levels between the subjects with and without genotype data in the German (Table S1A) and Japanese (Table S1B) cohorts. Click here for file

Additional file 2: Table S2 Comparisons of the genotype distributions of single nucleotide polymorphisms in *SFTPD* gene between the German and Japanese cohorts. Click here for file
