# Supplementary material for: Differences in serum SP-D levels between German and Japanese subjects are associated with SFTPD gene polymorphisms
Source: BMC Med Genet. 2014 Jan 8;15:4. doi: 10.1186/1471-2350-15-4 (PMC3893448; doi:10.1186/1471-2350-15-4)
Supplement: Additional file 1: Table S1 — Comparisons of the baseline characteristics including serum SP-A and SP-D levels between the subjects with and without genotype data in the German (Table S1A) and Japanese (Table S1B) cohorts. [file 1471-2350-15-4-S1.pdf]

Table S1A - Characteristics of German subjects

	with genotype data	without genotype data	
**German patients with IIPs**			
Number of the subjects	102	36	
Age (years)	67.3 ± 1.0	67.7 ± 1.6	0.986
Gender (male / female)	64 (62.7%) / 38 (37.3%)	24 (66.7%) / 12 (33.3%)	0.674
Smoking (Cu / Ex / Non / ND)	10 (9.8%) / 35 (34.3%) / 51 (50.0%) / 6 (5.9%)	4 (11.1%) / 19 (52.8%) / 13 (36.1%) / 0 (0.0%)	0.194
VC (percent predicted)	69.4 ± 2.2	72.8 ± 2.8	0.036
DL_{CO} (percent predicted)	47.1 ± 2.1	49.4 ± 2.5	0.316
Diagnostic category (IPF / NSIP)	71 (69.6%) / 31 (30.4%)	23 (63.9%) / 13 (36.1%)	0.527
Serum SP-A (ng/ml)	79.5 ± 3.3	79.0 ± 8.5	0.253
Serum SP-D (ng/ml)	376.0 ± 23.2	365.4 ± 42.2	0.466
**German Healthy subjects**			
Number of the subjects	37	128	
Age (years)	42.9 ± 2.3	34.7 ± 0.9	0.001
Gender (male / female)	17 (45.9%) / 20 (54.1%)	43 (33.6%) / 85 (66.4%)	0.169
Smoking (Cu / Ex / Non / ND)	4 (10.8%) / 2 (5.4%) / 17 (45.9%) / 14 (37.9%)	37 (28.9%) / 18 (14.1%) / 73 (57.0%) / 0 (0.0%)	0.315
Serum SP-A (ng/ml)	31.1 ± 2.9	29.3 ± 1.2	0.942
Serum SP-D (ng/ml)	62.2 ± 8.6	59.1 ± 2.2	0.443

Table S1B - Characteristics of Japanese subjects

	with genotype data	without genotype data	
**Japanese patients with IIPs**			
Number of the subjects	63	31	
Age (years)	67.8 ± 1.3	68.3 ± 1.3	0.961
Gender (male / female)	43 (68.3%) / 20 (31.7%)	21 (67.7%) / 10 (32.3%)	0.960
Smoking (Cu / Ex / Non / ND)	10 (15.9%) / 30 (47.6%) / 23 (36.5%) / 0 (0.0%)	1 (3.2%) / 16 (51.6%) / 12 (38.7%) / 2 (6.5%)	0.233
VC (percent predicted)	73.2 ± 2.6	67.0 ± 4.9	0.101
DL_{CO} (percent predicted)	45.2 ± 2.1	43.8 ± 3.0	0.912
Diagnostic category (IPF / NSIP)	37 (58.7%) / 26 (41.3%)	24 (77.4%) / 7 (22.6%)	0.074
Serum SP-A (ng/ml)	78.7 ± 6.6	91.8 ± 13.8	0.442
Serum SP-D (ng/ml)	308.6 ± 42.8	331.4 ± 39.3	0.955
**Japanese Healthy subjects**			
Number of the subjects	275	0	
Age (years)	49.8 ± 0.4	NA	
Gender (male / female)	227 (82.5%) / 48 (17.5%)	NA	
Smoking (Cu / Ex / Non / ND)	82 (29.8%) / 62 (22.6%) / 131 (47.6%) / 0 (0.0%)	NA	
Serum SP-A (ng/ml)	29.4 ± 0.9	NA	
Serum SP-D (ng/ml)	39.9 ± 1.6	NA	

Data are shown as mean ± SEM.

Statistical significance was tested by Mann–Whitney *U*-test or Chi-square test.

Cu, current smoker; Ex, ex-smoker; Non, non-smoker; ND, no data; NA, not available, IIPs, idiopathic interstitial pneumonias; VC, vital capacity; DL_{CO}, diffusing capacity of the lung for carbon monoxide; IPF, idiopathic pulmonary fibrosis; NSIP, nonspecific interstitial pneumonia.
