# Supplementary material for: Differences in serum SP-D levels between German and Japanese subjects are associated with SFTPD gene polymorphisms
Source: BMC Med Genet. 2014 Jan 8;15:4. doi: 10.1186/1471-2350-15-4 (PMC3893448; doi:10.1186/1471-2350-15-4)
Supplement: Additional file 2: Table S2 — Comparisons of the genotype distributions of single nucleotide polymorphisms in SFTPD gene between the German and Japanese cohorts. [file 1471-2350-15-4-S2.pdf]

Table S2 - Genotype distributions of single nucleotide polymorphisms in *SFTPD* gene – German vs Japanese –

		**Genotype distributions**		
**rs721917**	total	C/C	T/C	T/T
German	139	34 (24.5%)	58 (41.7%)	47 (33.8%)
Japanese	338	114 (33.7%)	166 (49.1%)	58 (17.2%)
Chi-square test				$p < 0.001$
**rs1998374**	total	C/C	T/C	T/T
German	139	3 (2.2%)	23 (16.5%)	113 (81.3%)
Japanese	338	63 (18.7%)	160 (47.3%)	115 (34.0%)
Chi-square test				$p < 0.001$
**rs2243639**	total	C/C	T/C	T/T
German	139	56 (40.3%)	60 (43.2%)	23 (16.5%)
Japanese	338	168 (49.7%)	141 (41.7%)	29 (8.6%)
Chi-square test				$p = 0.022$
**rs3088308**	total	A/A	A/T	T/T
German	139	118 (84.9%)	21 (15.1%)	0 (0.0%)
Japanese	338	324 (95.9%)	14 (4.1%)	0 (0.0%)

Each of German and Japanese cohorts includes both patients and healthy subjects.
